# Central Hemodynamic Adjustments during Post-Exercise Hypotension in Hypertensive Patients with Ischemic Heart Disease: Concurrent Circuit Exercise versus High-Intensity Interval Exercise. A Preliminary Study

**DOI:** 10.3390/jcm10245881

**Published:** 2021-12-15

**Authors:** Giuseppe Caminiti, Ferdinando Iellamo, Marco Alfonso Perrone, Valentino D’Antoni, Matteo Catena, Vincenzo Manzi, Valentina Morsella, Alessio Franchini, Maurizio Volterrani

**Affiliations:** 1 Cardiology Rehabilitation Unit, S. Raffaele IRCCS, 00163 Rome, Italy; giuseppe.caminiti@sanraffaele.it (G.C.); iellamo@uniroma2.it (F.I.); valentino.dantoni@sanraffaele.it (V.D.); matteo.catena.94@gmail.com (M.C.); valentina.morsella@sanraffaele.it (V.M.); franchini.fkt@gmail.com (A.F.); maurizio.volterrani@sanraffaele.it (M.V.); 2 Department of Clinical Sciences and Translational Medicine, University of Rome Tor Vergata, 00133 Rome, Italy; 3 Dipartimento di Scienze Umanistiche, Università Telematica Pegaso, 80132 Naples, Italy; vimanzi@yahoo.com

**Keywords:** post-exercise hypotension, ischemic heart disease, exercise, hypertension, cardiac rehabilitation

## Abstract

Concurrent aerobic plus resistance exercise (RAE) and high-intensity interval exercise (HIIE) are both effective at inducing post-exercise hypotension (PEH) in patients with hypertension. However, central hemodynamic changes associated with PEH in hypertensive subjects with underlying ischemic heart disease (IHD) have been poorly investigated. The study aim was to compare the acute effects produced by these two exercise modalities on left ventricular diastolic function and left atrial function. Twenty untrained male patients with a history of hypertension and IHD under stable pharmacological therapy were enrolled. Each patient underwent three exercise sessions: RAE, HIIE and a control session without exercise, each lasting 45 min. An echocardiography examination was performed before and between 30 min and 40 min from the end of the exercise sessions. Following the exercise sessions, BP values decreased in a similar way in RAE and HIIE and were unchanged after the control session. Compared to pre-session, the ratio between early filling velocity (E) and mitral annulus early diastolic velocity (E’). E/E’ increased after HIIE and remained unchanged after both RAE and control sessions (between-sessions *p* 0.002). Peak atrial longitudinal strain (PALS) increased slightly after RAE (+1.4 ± 1.1%), decreased after HIIE (−4.6 ± 2.4%) and was unchanged after the control session (between-sessions *p* 0.03). Peak atrial contraction strain (PACS) was mildly increased after RAE, was reduced after HIIE and was unchanged after the control session. Atrial volume was unchanged after both exercise sessions. Left ventricular and left atrial stiffness increased significantly after HIIE, but remained unchanged after the RAE and control sessions. Stroke volume and cardiac output increased after RAE, decreased after HIIE, and were unchanged after the control session. In conclusion, single session of RAE and HIIE brought about similar PEH in hypertensive subjects with IHD, while they evoked different central hemodynamic adjustments. Given its neutral effects on diastolic and atrial functions, RAE seems more suitable for reducing blood pressure in hypertensive patients with IHD.

## 1. Introduction

Physical exercise is a well-established non-pharmacological treatment for patients with hypertension and with ischemic heart disease (IHD) [1]. Continuous aerobic exercise is the most commonly used modality in the rehabilitation of these patients; alternatively, high-intensity interval exercise (HIIE) and concurrent resistance and aerobic exercise (RAE) are employed with growing frequency, since they present a wide range of benefits that include greater impact on oxygen consumption and muscle strength, as well as additive effects on metabolism and hormonal profile [2,3,4,5,6]. When exercise is used for treating hypertension, the reduction of blood pressure (BP) below resting pre-exercise levels after a single session, called post-exercise hypotension (PEH), is a clinically relevant phenomenon, since it has been suggested that it may be useful to predict individual responsiveness to BP decrease after a training period [7]. Some recent studies comparing the effectiveness of acute HIIE and RAE to elicit PEH in hypertensive patients showed BP reductions of similar magnitude following these two exercise modalities, at least when they are performed by untrained patients [8,9]. In previous trials 3–6 months of HIIE and RAE proved to be safe and effective in elderly subjects and in patients with cardiovascular diseases; these patients were able to improve their exercise tolerance without harming the hemodynamic profile [10,11,12,13,14]. However central hemodynamic changes that occur early with PEH after a single session of HIIE or RAE in patients with hypertension and underlying IHD have been poorly investigated. In this group of patients, the concomitant presence of hypertension and myocardial ischemia contributes over time to increase left ventricular (LV) stiffness and to generate diastolic dysfunction. Diastolic dysfunction, in turn, worsens LV filling and is a strong predictor of cardiovascular events and heart failure [15,16]. Therefore, prescribing physical exercise for reducing BP and improving exercise tolerance without harming diastolic function or even, possibly, improving it could be a desirable goal for these patients. 

The aim of this study was to compare the central hemodynamic responses occurring after a single session of RAE and HIIE in patients with hypertension and IHD in order to establish which exercise modality is more suitable for reducing BP in these patients. The first endpoint of the study was changes in the ratio between early filling velocity (E) and mitral annulus early diastolic velocity (E’). We hypothesized that the two exercise modalities would have similar effects on the E/E’ ratio. Secondary endpoints were changes in left atrial (LA) and LV systolic functions.

## 2. Methods

The present research was a sub-study of the AtrialSTRain after EXercise (ASTREX) trial and included the first 20 patients enrolled in the ASTREX. The ASTREX trial compares acute and long-term effects of interval training versus combined training on echocardiography parameters in hypertensive male patients with IHD undergoing cardiac rehabilitation. It was approved by the internal ethical committee of S. Raffaele IRCCS Rome, and registered to Clinical trial.gov (registration number: NCT04763629), and it is still ongoing. In the present study, in addition to the original design of the ASTREX trial, after performing the first exercise session of their training program (according to their group allocation in the ASTREX) patients were asked to perform a single session of the exercise modality of the opposite arm, and a control session without exercise. The following inclusion criteria were adopted: history of hypertension and IHD; sedentary patients not being enrolled in exercise training programs in the previous six months; male gender; age over 45 years. The exclusion criteria were: secondary hypertension; clinical BP levels exceeding 160/100 mmHg; significant heart valve diseases; hypertrophic cardiomyopathy; signs and/or symptoms of myocardial ischemia during an initial ergometric test; uncontrolled arrhythmia; neurological and/or orthopaedic conditions contraindicating or limiting exercise; significant chronic obstructive pulmonary disease (FEV1 < 50%) or symptomatic peripheral arterial disease. For the purpose of the study, hypertensive patients were defined as those who had a history of hypertension and/or were being treated with anti-hypertensive drugs at the enrolment. The diagnostic criteria for IHD were: previous myocardial infarction; a history of percutaneous coronary interventions and/or coronary artery bypass graft.

### 2.1. Experimental Protocol

This study had a cross-over design. Each participant performed three experimental sessions: a HIIE session, a RAE session and a control session without any exercise. The order of the experimental sessions was randomly assigned by means of computer software. The experimental sessions were performed in the morning, between 9:30 and 11:00, and lasted 45 min. Each patient performed the experimental sessions on different days. Patients were asked not to smoke, to have a light breakfast at least 2 h before the start of the session and to take their morning drugs regularly. To establish a baseline, in a preliminary visit, all patients performed a cardiopulmonary test in order to rule out exercise-induced ischemia and other contraindications to exercise and to establish training intensities and the assessment of maximal voluntary contraction of different muscle groups involved in the resistance portion of the concurrent session. The cardiopulmonary test was performed on a treadmill and a standard Bruce protocol was adopted in each patient. At the beginning of each session, participants rested in the seated position for 15 min and underwent standardized BP measurements in the dominant arm, in duplicate, using a calibrated oscillometric automatic device (OMRON, Healthcare Inc., Milan, Italy). Measures of BP were then assessed 15, 30 and 60 min after the end of the exercise sessions. During the HIIE session patients walked on a treadmill. They performed three peaks of high-intensity exercise, each one lasting 5 min at 80–95% of VO_2_, spaced by three intervals of lower-intensity exercise performed at 50–60% of peakVO_2_, each one lasting 10 min. During the RAE session, patients walked for 25 min on a treadmill at 55–70% of peakVO_2_, then they performed resistance exercises with arms and legs, consisting of 2 sets of 10 repetitions at 60% of 1 repetition maximum (RM), with 2 min rest between sets. Resistance training consisted of the following exercises: leg press and extension, shoulder press, chest press, low row and vertical traction (Technogym Wellness System, Technogym, Cesena, Italy). During the control session the patients stayed at rest, sitting for 45 min between the two echocardiography assessments.

### 2.2. Echocardiography

All echocardiographic examinations were made using an Acuson SC 2000 Prime ultrasound system (Siemens) with a 4.0 MHz transducer operated by one experienced sonographer who was blinded to the type of experimental sessions performed by patients. Left ventricular end-diastolic volume (LVEDV) and end-systolic volume (LVESV) were calculated from the apical two and four chamber using modified Simpson’s method, which was used to calculate stroke volume (SV) as EDV–ESV, cardiac output (CO) as HR × SV, and ejection fraction (EF) as EF = (EDV−ESV)/EDV. The E/A ratio represents the ratio of peak left ventricle filling velocity in early diastole (E wave) to that in late diastole, during atrial contraction (A wave). The LV E/E’ ratio was calculated as the ratio between E wave velocity and mean lateral and septal LV E’ wave velocities. Colour tissue Doppler tracings were obtained with the range gate placed at the lateral mitral annular segments in the four-chamber view. Peak systolic LV longitudinal strain and strain rates were assessed using standard 2D apical four-chamber, two-chamber and three-chamber views using speckle-tracking analysis. Global longitudinal strain (GLS) was determined by averaging all values of the 18 segments of the three views. Measurement of LA strain was obtained from the 4-chamber and 2-chamber views. The software generates the longitudinal strain curves for each segment and a mean curve of all segments. Peak atrial longitudinal strain (PALS) was measured at the end of the reservoir phase (positive peak during LV systole). Peak atrial contraction strain (PACS) was measured just before the start of the active contractile phase (positive peak during early diastole). LV stiffness was calculated as E/E’/LVEDV [17] whilst LA stiffness was calculated as E/E’/PALS [18]. Diastolic dysfunction was defined according to ASE/ESC recommendations [19].

### 2.3. Statistical Analysis

Since we did not find previous direct comparisons between HIIE and RAE in the literature, we were not able to calculate the study sample size; therefore, this research has been conceived as a pilot study. Data are expressed as mean ± SD. The assumption of normality was checked using the Shapiro–Wilk hypothesis test. Pre- and post- exercise data of normally distributed variables were assessed using repeated measure two-way ANOVA and Bonferroni corrections for *post hoc* testing; while non-normally distributed variables were assessed using the Kruskal–Wallis test and Bonferroni corrections for *post hoc* testing. The level of significance was set at *p* < 0.05. Data were analysed using SPSS software (version 20.0 IBM Corp., Amonk, New York, NY, USA).

## 3. Results

The mean age of the sample was 67.3 ± 11.4 years; mean body mass index was 26.4 ± 5.2. Eleven out of 20 (55%) patients had a previous myocardial infarction, thirteen (65%) had undergone percutaneous coronary interventions, and nine (45%) had undergone coronary artery bypass graft. Five patients (25%) had type-2 diabetes. Eleven (55%) were ex-smokers and there were no current smokers. All patients were taking antiplatelet drugs, statins, betablockers and angiotensin-converting enzyme inhibitors or angiotensin receptor blockers. Seven patients (35%) were taking calcium channel blockers and two (10%) were taking transdermic nitrates. The average number of anti-hypertensive drugs was 2.6 ± 1.3. All patients included in this study completed the study protocol and no side effects occurred during the two exercise sessions. At baseline, 15 patients had grade I and five had grade II diastolic dysfunction.

**Figure 1:**
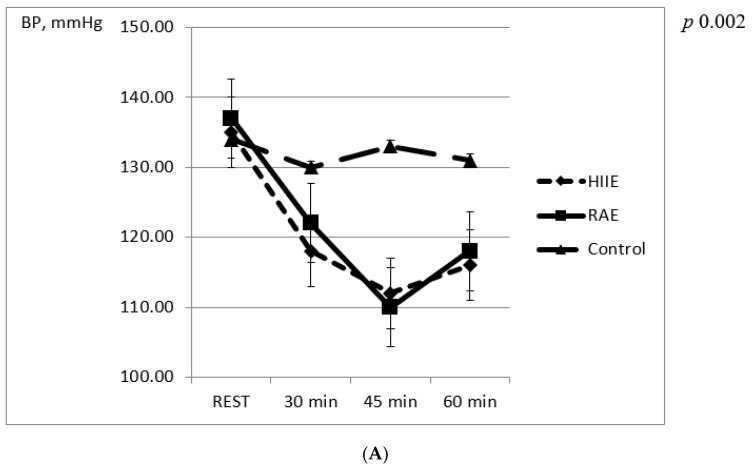

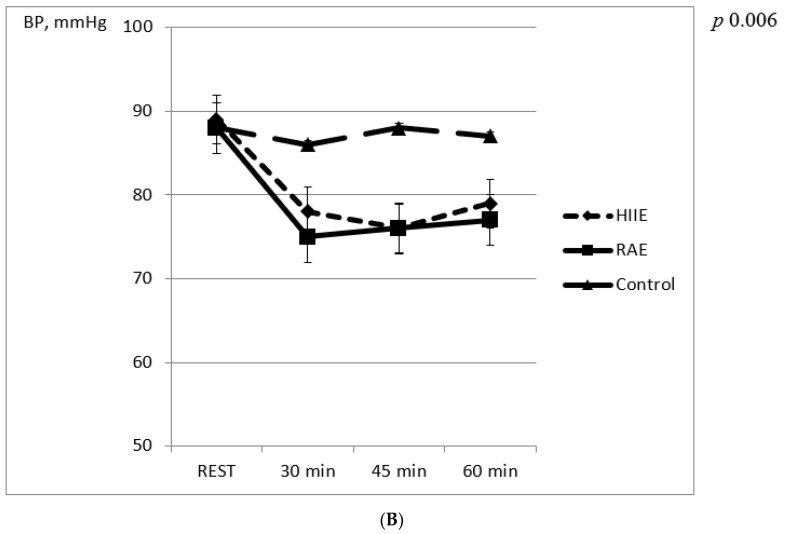
(**A**) Changes in systolic blood pressure in the first hour after experimental sessions. (**B**) Changes in diastolic blood pressure in the first hour after experimental sessions.

Post-exercise systolic and diastolic BP decreased significantly compared to baseline values after both sessions (Figure 1). There were no significant differences between RAE and HIIE at 15, 30 and 60 min after the exercise sessions. BP was unchanged in the control group. 

### 3.1. LV Diastolic Function and LV Stiffness

No changes in the degree of diastolic dysfunction occurred after RAE; conversely, 5 patients remained grade I, 9 displayed grade II and 6 displayed grade III diastolic dysfunction after HIIE. No changes in diastolic function occurred after the control session. The E/E’ ratio increased slightly after the RAE session, and presented a significant increase following the HIIE session (between-session Δ +4.8; F = 3.8; *p* 0.002) (Table 1); E’ wave decreased after the HIIE session and remained unchanged after RAE. The E/E’ ratio and E’ wave remained unchanged in the control group. There was a decrease in LV diastolic volume after both RAE and HIIE, reaching statistical significance only in the HIIE group (between-session Δ −4 mL; F = 1.3; *p* 0.14).

### 3.2. Left Atrial Function and LA Stiffness

There was a mild increase in PALS after the RAE session, but it did not decrease significantly after HIIE and was unchanged after a control session (between-session Δ = +6; F = 3.2; *p* 0.03). There was also a mild increase in PACS after RAE, but it was unchanged after HIIE and control sessions (between-session Δ = +2.4; F = 1.7; *p* 0.09). Atrial stiffness increased significantly after HIIE sessions but remained unchanged after RAE and control sessions. Atrial volume was unchanged after both exercise sessions. 

### 3.3. Left Ventricular Systolic Function

CO and SV showed a non-significant increase after RAE, were unchanged after control sessions, and presented a non-significant decrease after HIIE (between-sessions CO: Δ = −0.4 ± 1.5 L/min; F = 1.6, p 0.09; SV: Δ = 9 ± 1.5 mL; F = 1.1; p 0.11). GLS and EF were unchanged after both RAE and HIIE sessions. No changes occurred in the control group regarding CO and indices of LV systolic function.

## 4. Discussion

The most important finding of this study is that single sessions of RAE and HIIE exerted different effects on E/E’ ratio as well as on other hemodynamic parameters in untrained hypertensive subjects with underlying IHD. In particular, it was observed that after RAE there were no changes in LV stiffness, E/E’ ratio or SV compared to pre-exercise, while CO presented an insignificant increase. Conversely HIIE was associated with a significant increase in the E/E’ ratio, while SV and CO showed a non-significant decrease. It is thought that HIIE, by increasing the degree of diastolic dysfunction, caused a decrease in LV filling and ultimately contributed to preventing an adequate increase in SV and CO. Our findings, despite being in need of further confirmation via larger trials, appear to be in line with a previous study in a similar population, which underlined the role of diastolic function in determining CO irrespective of LV systolic function [20]. While long-term training interventions with different exercise modalities have been associated with positive or neutral effects on diastolic function [10,11,12,13,14,21,22], a single bout of intense or prolonged exercise can induce short-term alterations in LV diastolic function [23,24]. These unfavourable changes are usually transient, with restoration of pre-exercise function typically observed after 24–48 h of recovery [23]. Therefore, taking into consideration the present results, it is thought that the HIIE protocol adopted in this study was too strenuous for untrained patients with hypertension and IHD. Interestingly, despite the fact that HIIE and RAE evoked different central hemodynamic adaptations, they produced a similar magnitude of PEH. The data from the present study suggest that central hemodynamic changes were not involved in the onset of PEH shortly after RAE and that in this case the fall in BP was probably due to peripheral vasodilation; instead, central hemodynamic changes concurred with the onset of PEH after HIIE. The mechanisms underlying PEH have been widely investigated, but there is little data on acute hemodynamic changes occurring after RAE and HIIE, particularly in patients with cardiac diseases. The acute hemodynamic response to RAE has been assessed in healthy subjects [25] and in patients with hypertension [26] with variable results produced. In partial disagreement with our findings, another study [26] showed that compared to pre-session measurements, HR increased, systemic vascular resistance remained unchanged and CO was reduced. Regarding hemodynamic changes occurring after HIIE, the data from the present study differ from those obtained in a previous study, [27], which evaluated patients with mild non-ischemic HF using cardiac magnetic resonance imaging before and after a session of HIIE. The authors observed that LV systolic and diastolic functions were unchanged immediately after HIIE compared to pre-exercise. Clearly, the type of population – in particular the presence or lack of cardiac diseases, the exercise protocols adopted and the imaging techniques utilized may affect the results of these studies [28]. Moreover, in patients with chronic hypertension and IHD, such as those included in this study, an upregulation of the sympathetic nervous system (SNS) is expected which, on the one hand, results in dysfunctional baroreceptor and chemoreceptor reflexes, and on the other hand, leads to the outflow of augmented catecholamines to the heart, vessels and skeletal muscles [29]. Ultimately, this abnormal SNS activation deeply affects the central and peripheral cardiovascular responses to exercise [30]. Additionally, the use of anti-hypertensive medication may have affected the magnitude of PEH and its underlying mechanisms [31]. In this study, changes in opposite directions of post-exercise HR were observed: compared to baseline resting values, HR increased after RAE but decreased after HIIE. Changes in HR might have occurred as a consequence of different acute modulatory effects produced by RAE and HIIE on the autonomic nervous system. We were not able to verify this hypothesis based on the results of this study due to the lack of direct assessment of sympathovagal balance; however, an increased cardiac vagal activity after HIIE has been previously found [32,33], while RAE has been linked to an increased cardiac sympathetic activation [24] that was mostly related to the resistance component of the exercise session [34]. In this study, HIIE and RAE also had different effects on atrial function: PALS and PACS were slightly increased after RAE, but decreased after HIIE. The reduction of both the reservoir and booster functions of LA may have been another factor, in addition to the increase in LV stiffness, which contributes to worse LV diastolic filling, to decreased LV preload and, ultimately, to the post-exercise reduction of SV after HIIE. Acute exercise-induced changes on LA function have been investigated, mostly in animal models [35] and healthy subjects [36], where increases in both LA reservoir and booster function during exercise have been shown. However, that exercise-mediated increase in atrial function seems to be lost in pathological conditions. In patients with heart failure, one study found that during a ramp incremental test, LA reservoir and booster functions were impaired both at peak exercise and during the recovery phase when compared to healthy controls [37]. To date, the long-term effects on atrial function produced by various exercise modalities have been assessed mainly in elite athletes [36] and there is still little data regarding patients with cardiovascular conditions. In an uncontrolled study performed on post-AMI patients [38], after six weeks of cardiac rehabilitation LA strain improved, but all other parameters, including atrial volume and diastolic function, were unchanged. More recently, a similar result was obtained with hypertensive patients undergoing cardiac rehabilitation [39]. We did not find any previous data on acute changes of LA function occurring after a single session in hypertensive patients with IHD. 

This study presents several limitations. The most important limitation is the small sample size; further larger studies on this topic are needed in order clarify central adaptations induced by different exercise modalities. All hemodynamic parameters were measured or calculated by echocardiography and the study therefore lacks comparison with invasive hemodynamic assessment through cardiac catheterization. However, echocardiography is a widely used and accepted method for monitoring the acute and long-term effects of exercise on cardiac parameters [9,11,13,25]. All patients were untrained; we cannot rule out that training status may modify central hemodynamic adjustments occurring after RAE and HIIE. In this study we did not standardize in a rigorous way participants’ meals in the days preceding the exercise sessions, and this could be a potential confounding factor given that certain foods may change BP values. However, experimental sessions were performed in the same period of the morning, patients were asked to refrain from smoking cigarettes, and they were only allowed to consume a light breakfast at least 2 h before the beginning of the sessions. BP was measured on the dominant arm exclusively, and we are therefore unable to exclude potential differences with the contralateral arm; however the cross-over design of this study reduces the weight of this potential confounding factor. Post-exercise echocardiography was performed from 30 to 40 min from the end of exercise sessions and was not repeated further, therefore we cannot establish how long post-exercise hemodynamic changes lasted. Similarly, BP was not measured beyond the first hour, as a result of which we have no information on BP changes occurring afterwards. We did not assess autonomic nervous system activity or peripheral vascular resistance directly, therefore we are unable to provide a full description of post-exercise hemodynamic changes related to PEH after RAE and HIIE sessions; however, the main objective of this study was to identify which exercise modality was associated with a better diastolic profile and was more suitable for our patients. Only men were included in this study, therefore our results cannot be generalized to females. 

In conclusion, single sessions of RAE and HIIE determined similar PEHs in untrained hypertensive patients with IHD. PEH after HIIE was associated with a worsened LA function and LV diastolic function; on the contrary, RAE did not affect LV and LA stiffness and did not generate diastolic dysfunction. Therefore, RAE seems to be a more suitable exercise modality for untrained hypertensive patients with IHD and may be preferable at the beginning of a training program. 

## 5. Conclusions

In this study, single sessions of RAE and HIIE determined similar PEH in untrained hypertensive subjects with IHD; however, they evoked different central hemodynamic adjustments. Given its neutral effects on diastolic and atrial functions, RAE seems to be a more suitable exercise modality for reducing blood pressure in hypertensive patients with IHD, at least when they start a training program. Given the small sample size, the findings of this study need to be confirmed in further larger trials. 

## Figures and Tables

**Table 1:** Echocardiography parameters before and after experimental sessions.

	Pre-RAE	Post-RAE	Pre-HIIE	Post-HIIE	Controls (T0)	Controls (T1)	Between-Group *p*
**LV Function**							
HR, bpm	62.8 ± 13.4	67.5 ± 11.2	57.2 ± 20.6	55.9 ± 19.3	60.8 ± 15.2	60.4 ± 17.7	0.238
EDV, mL	164.3 ± 37.3	154.7 ± 48.5	162.6 ± 44.2	148.6 ± 39.7	160.3 ± 46.2	162.3 ± 40.4	0.142
ESV, mL	74.1 ± 18.5	73.2 ± 13.9	72.9 ± 19.4	62.5 ± 17.3	74.5 ± 21.2	72.5 ± 15.8	0.190
SV, mL	80.1 ± 20.6	82.4 ± 26.2	81.7 ± 21.5	74.1 ± 28.2	80.7 ± 17.5	79.3 ± 19.4	0.117
CO, L/min	5.3 ± 1.8	5.5 ± 1.3	5.3 ± 2.5	5.1 ± 1.8	5.3 ± 2.1	5.2 ± 1.4	0.092
GLS, %	−15.6 ± 3.7	−15.0 ± 2.9	−15.7 ± 3.1	−15.2 ± 4.4	−15.5 ± 5.0	−15.3 ± 3.6	0.277
EF, %	52.4 ± 6.6	53.1 ± 8.1	51.5 ± 7.8	52.1 ± 8.3	52.4 ± 6.9	51.9 ± 9.0	0.314
E, cm/s	69.0 ± 21.3	64.3 ± 24.1	69.5 ± 18.7	61.8 ± 15.6	69 ± 16.0	68 ± 17.1	0.289
A, cm/s	68.5 ± 16.8	68.3 ± 18.3	70.8 ± 19.5	71.1 ± 16.2	70.6 ± 19.2	69.4 ± 21.0	0.332
E’, cm/s	9.1 ± 1.5	8.7 ± 2.2	9.5 ± 1.9	5.5 ± 1.4 *	9.4 ± 2.0	9.3 ± 1.8	0.085
E/E’	7.5 ± 1.7	8.1 ± 2.4	7.6 ± 1.1	12.2 ± 1.6 *	7.6 ± 2.2	7.8 ± 1.9	0.002
LV stiffness	0.045 ± 0.7	0.052 ± 0.9	0.046 ± 0.6	0.082 ± 0.4 *	0.047 ± 0.2	0.048 ± 0.8	0.013
**LA Function**							
PALS, %	37.8 ± 11.0	39.4 ± 6.7	35.8 ± 9.3	31.2 ± 10.5	37.8 ± 12.5	37.2 ± 11.3	0.032
PACS, %	18.6 ± 2.1	20.9 ± 2.6	15.6 ± 2.3	15.5 ± 1.8	18.6 ± 2.6	18.9 ± 2.8	0.096
LA stiffness	0.20 ± 0.08	0.22 ± 0.04	0.21 ± 0.07	0.38 ± 0.06 *	0.20 ± 0.04	0.20 ± 0.05	0.083
LAVI, mL/m^2^	32.4 ± 3.6	33.0 ± 4.1	32.7 ± 4.0	33.5 ± 3.6	32.0 ± 5.9	32.3 ± 4.6	0.302

EDV = end diastolic volume; ESV = end systolic volume; SV = stroke volume; CO = cardiac output; GLS = global longitudinal strain; EF = ejection fraction; E/E’= ratio between early filling velocity (E) and mitral annulus early diastolic velocity (E’). PALS = peak atrial longitudinal strain; PACS = peak atrial contraction strain; LAVI = left atrial volume index. * *p* < 0.05 (pre-exercise vs post-exercise). Statistical tests: two-way ANOVA and Bonferroni corrections for normally distributed parameters; Kruskal–Wallis test and Bonferroni corrections for non-normally distributed parameters.

## Data Availability

The data presented in this study are available on request from the corresponding author.
